# Cardiovascular risk screening of patients with serious mental illness or use of antipsychotics in family practice

**DOI:** 10.1186/s12875-020-01225-7

**Published:** 2020-07-29

**Authors:** Kirsti M. Jakobs, Anne Posthuma, Wim J. C. de Grauw, Bianca W. M. Schalk, Reinier P. Akkermans, Peter Lucassen, Tjard Schermer, Willem J. J. Assendelft, Marion J. C. Biermans

**Affiliations:** Department of Primary and Community Care (117-ELG), Radboud University Medical Centre, Radboud Institute for Health Sciences, PO Box 9101, 6500 HB Nijmegen, The Netherlands

**Keywords:** Serious mental illness, Severe mental illness, Psychotic disorders, Cardiovascular risk, Antipsychotic agents, Screening, Family practice

## Abstract

**Background:**

Patients with serious mental illness (SMI) and patients on antipsychotics (AP) have an elevated risk for cardiovascular diseases. In the Netherlands, the mental healthcare for these patients is increasingly taken care of by family practitioners (FP) as a result of a shift from secondary to primary care. Therefore, it is essential to increase our knowledge regarding the characteristics of this patient group and the (somatic) care provided by their FPs. The aim was to examine the rate of cardiovascular risk screening in patients with SMI or the use of AP in family practice.

**Methods:**

We performed a retrospective cohort study of 151.238 patients listed in 24 family practices in the Netherlands.

From electronic medical records we extracted data concerning diagnoses, measurement values of CVR factors, medication and frequency of visits over a 2 year period. Primary outcome was the rate of patients who were screened for CVR factors. We compared three groups: patients with SMI/AP without diabetes or CVD (SMI/AP-only), patients with SMI/AP and diabetes mellitus (SMI/AP + DM), patients with SMI/AP and a history of cardiovascular disease (SMI/AP + CVD). We explored factors associated with adequate screening using multilevel logistic regression.

**Results:**

We identified 1705 patients with SMI/AP, 834 with a SMI diagnosis, 1150 using AP. The screening rate for CVR in the SMI/AP-only group (*n* = 1383) was adequate in 8.5%. Screening was higher in the SMI/AP − +DM (*n* = 206, 68.4% adequate, OR 24.6 (95%CI, 17.3–35.1) and SMI/AP + CVD (*n* = 116, 26.7% adequate, OR 4.2 (95%CI, 2.7–6.6). A high frequency of visits, age, the use of AP and a diagnosis of COPD were associated with a higher screening rate. In addition we also examined differences between patients with SMI and patients using AP without SMI.

**Conclusion:**

CVR screening in patients with SMI/AP is performed poorly in Dutch family practices. Acceptable screening rates were found only among SMI/AP patients with diabetes mellitus as comorbidity. The finding of a large group of AP users without a SMI diagnosis may indicate that FPs often prescribe AP off-label, lack information about the diagnosis, or use the wrong code.

## Background

Both a diagnosis of serious mental illness (SMI) and the use of antipsychotics (AP) are associated with an elevated cardiovascular risk. SMI incorporates schizophrenia, bipolar disorder and other psychotic disorders [1]. People with SMI have an 8–20 years shorter life expectancy compared to the general population [2, 3], which is mainly caused by CVD [4–6]. The etiology of the increased risk for CVD in patients with SMI is multifactorial, including high levels of smoking and other substance misuse, poor dietary intake, inadequate amount of exercise, less access to medical care, obesity, diabetes and adverse effects of AP [6–16]. The use of AP increases the risk of CVD via metabolic pathways involving weight gain, glucose intolerance, dyslipidemia and can cause cardiac toxicity [4, 17–19]. Patients get AP prescribed for SMI, but a growing group receives AP prescriptions off-label. Main indications for off-label prescription are mood disorders, anxiety disorders, insomnia and agitation [20].

Guidelines [21–24] and medicine agencies [25, 26] recommend annual screening for cardiovascular risk factors in patients with SMI and in all patients using AP. Unfortunately, assessment of and treatment for CVR is often performed poorly [8, 27–34] due to both patient [8, 16, 32–34] and physician-related [8, 16] factors and the lack of collaboration between family physicians (FP) and psychiatrists [5, 32, 35]. In addition, some psychiatrists lack the knowledge and competence required for diagnosing and treating CVR factors [16, 32].

In the UK, a SMI register has been established. However, the monitoring of CVR for patients receiving AP without having SMI remains unaccounted for. As a result of a governmentally regulated shift from secondary to primary care, mental healthcare for patients with SMI and/or receiving AP (SMI/AP) in the Netherlands and in the UK is increasingly under direction of FPs [36–38]. This creates an opportunity for the patients to receive CVR screening in the chronic care programs and also provides financial incentive for the FP. FPs can be of added value because CVR prevention is their daily task in high risk patients. It also introduces the question of responsibility for the CVR screening in relation to the medication use. Therefore, it is essential to increase our knowledge regarding the (somatic) care provided by FPs for these patients.

The primary aim of our study is to examine the cardiovascular risk screening practice in patients with serious mental illness or those using anti-psychotics in family practice and to identify patient characteristics that are associated with the rate of screening.. We will describe a) the screening rate in SMI/AP patients without additional comorbidities, and compare this to b) the screening rate in a group of patients who have SMI/AP and an additional reason for CVR screening: diabetes and /or known cardiovascular morbidity. The first screening rate shows the task performed by FPs for reason of SMI/AP, the latter shows what can be achieved in primary care in this patient category, despite the earlier mentioned barriers.

## Methods

### Study design

This study is a retrospective cohort study of patients with SMI/AP in Dutch family practice.

### Study population and procedure

We followed the STROBE guidelines for reporting observational studies [39]. Our data were derived from a de-identified database, the Radboudumc Technology Center Health Data. This database contains Electronic Medical Records (EMRs) of family practices with information on patient demographics, diagnoses and symptoms, laboratory test results and drug prescriptions, number of visits (i.e. visits to the practice) along with characteristics of the family practices such as number of patients registered and geographical location. Drug prescriptions are coded according to the WHO Anatomical Therapeutic Chemical (ATC) Classification system [40]. Diagnoses and symptoms are coded according to the International Classification of Primary Care (ICPC) [41]. The database provides reliable data because in the Netherlands nearly all people are registered in a family practice over a long period of time, and FPs are used to classify each visit, using the ICPC system. The FP operates as a “gatekeeper” for secondary care and consequently medical specialists inform the FP about diagnosis and treatment [42]. However, electronic records for outpatient psychiatric visits in the Netherlands are separate from the FP’s system. Therefore, visits to a psychiatrist and data concerning CVR collected there were not included. We selected patients who have an indication for yearly assessment of CVR based on their psychiatric disorder or based on the use of antipsychotic medication or lithium*.*

We used data from 151.238 persons, who were listed in any of the 24 involved family practices, selected by region and availability of data from our FP database, between January 2013 and December 2014. We selected patients with (I) schizophrenia, affective psychosis, bipolar disorder or psychosis not otherwise specified (NOS) with a diagnose date prior to 1-1-2013 or (II) at least two prescriptions of antipsychotics, or (III) a prescription of lithium, II and III prescribed for the first time before 1-7-2013. This date was chosen since we only had access to the prescription records in this defined study period. Patients were excluded if (I) aged younger than 18 years, (II) diagnosed with dementia, (III) diagnosed with delirium without the presence of a psychotic disorder, (IV) if they were not registered for more than 12 months in the selected family practice in our study period, since FPs usually assess a patient’s CVR profile once a year [43] and (V) diagnosed with rheumatoid arthritis, since CVR assessment in this patient category was introduced just before our study period and therefore could possibly confound our results [43, 44].

### Data collection

Patients with SMI/AP were divided into three groups (I) patients without another indication for yearly assessment of CVR according to the current FP guidelines [43] ‘SMI/AP-only group’. (II) Patients with SMI/AP and diabetes mellitus (DM), and thus an extra indication for CVR assessment ‘SMI/AP + DM group’. (III) Patients with SMI/AP and a history of a cardiovascular disease (CVD; i.e. stroke, angina pectoris, acute myocardial infarction, transient ischemic attack, intermittent claudication and aortic aneurysm), and therefore an extra indication for CVR assessments ‘SMI/AP + CVD group’. Patients with both DM and CVD at baseline were added to the SMI/AP + DM group because patients with DM are routinely part of a chronic care program that pro-actively invites patients for monitoring.

Our primary outcome measure was the screening rate of CVR, i.e. the proportion of patients in each subgroup that received screening for their CVR factors in the defined study period.

The CVR factors were selected as recommended in the Dutch FP guidelines (i.e. Body Mass Index (BMI), blood pressure, estimated Glomerular Filtration Rate (eGFR), smoking status, fasting glucose, lipid spectrum, use of alcohol, family history of cardiovascular disease) [43]. However, considering the observational nature of this study and screening criteria described in previous studies [29, 30], we included a broader range of assessments (Additional file 1: Appendix A1).

We divided the observed screening in three levels: adequate, moderate and insufficient, based on current Dutch FP guidelines [43]. The screening rate was considered ‘adequate’ when BMI, smoking status, blood pressure, glucose and cholesterol/HDL ratio were all recorded at least once during the observation period, since these are the assessments that are needed to assess the 10-year CVR of a patient and provides the indications for cardiovascular risk-lowering medication. The screening rate was considered ‘moderate’ when the assessment included BMI, smoking status and blood pressure, as these can be measured without a blood test. The screening rate was considered insufficient if it did not meet up to these requirements. A 2-year window was chosen to gain insight in the role and awareness of the FP in this matter. Since FPs usually invite their high-risk patients once a year, patients who were screened just over the 1-year time window because of a delay in their response, would be part of the unscreened group, which would underestimate the screening rate.

Moreover, we wanted to identify factors associated with any CVR screening (adequate or moderate). The following factors were studied: age, sex, type of psychiatric disease, use of antipsychotics, use of antidepressants, CVR medication (i.e. statins, blood pressure drugs and aspirin), COPD, abuse of alcohol or drugs, any records of social issues and frequency of visits. We selected ICPC-codes concerning diseases and social problems (see Additional file 1: Appendix A2) and prescription records of antidepressants for this purpose. The ATC codes of AP, lithium and antidepressants are listed in Additional file 1: Appendix A3. We also selected (home) visits and calculated the frequency of visits per year of each patient.

### Statistical analyses

Descriptive analyses were used to describe the patient characteristics and to provide insight in the screening rate in the three different patient groups. As a result of the hierarchical structure of the study (patients nested within practices), multilevel analyses (random intercept model) were performed that took into account the variability associated with each level of clustering. Logistic regression analysis was performed to test the differences in screening rates between the three groups.

In addition, for the SMI/AP-only group we investigated the patient characteristics from Table 1 that were associated with an adequate or moderate screening rate. First, we included characteristics for the multivariate model that were univariately associated with screening (*p* < 0.20). After that a backward regression analyses was performed with these characteristics. A *p*-value of < 0.05 was considered to be statistically significant, based on two-sided tests. A sub analysis was added to show if the results differ between two groups: patients who were included based on their diagnosis (SMI) and patients who use AP without a diagnosis that suits the use (Additional file 2). All analyses were carried out using IBM SPSS statistics 22.0.

**Table 1:** Comparison of patient characteristics

	SMI/AP-only group *n* = 1383	SMI/AP + DM group *n* = 206	SMI/AP + CVD group *n* = 116	Total sample *n* = 1705
**Sex** female	720 (52.1)	110 (53.4)	51 (44.0)	881 (51.7)
**Mean age** in years (SD)	44.9 (14.8)	58.5 (14.0)	61.8 (12.3)	47.7 (15.7)
**SMI diagnosis, total**	629 (45.5)	97 (47.1)	48 (41.4)	834 (48.9)
Schizophrenia	197 (14.2)	38 (18.4)	15 (12.9)	250 (14.7)
Affective Psychosis/Bipolar Disorder	217 (15.7)	34 (16.5)	24 (20.7)	275 (16.1)
Psychosis Not Otherwise Specified	307 (22.2)	28 (13.6)	11 (9.5)	346 (20.3)
**SMI/AP**				
SMI with AP	290 (21.0)	55 (26.7)	20 (17.2)	365 (21.4)
SMI without AP	399 (28.9)	42 (20.4)	28 (24.1)	469 (27.5)
AP without SMI	630 (45.6)	95 (46.1)	60 (51.7)	785 (46.0)
Only lithium	64 (4.6)	14 (6.8)	8 (6.9)	86 (5.1)
**Medication use**				
Antipsychotics, total	920 (66.5)	150 (72.8)	80 (69.0)	1150 (67.4)
Lithium	160 (11.6)	31 (15.0)	22 (19.0)	213 (12.5)
Antidepressants	558 (40.3)	91 (44.2)	58 (50)	707 (41.5)
CVR lowering medication	295 (21.3)	186 (90.3)	103 (88.8)	584 (34.3)
**Comorbidity**				
COPD	65 (4.7)	25 (12.1)	23 (19.8)	113 (6.6)
Alcohol abuse	68 (4.9)	19 (9.2)	12 (10.3)	99 (5.8)
Tobacco abuse	233 (16.8)	83 (40.3)	42 (36.2)	358 (21.0)
Drug abuse	101 (7.3)	3 (1.5)	2 (1.7)	106 (6.2)
**Number of visits FP /year**				
0	393 (28.4)	46 (22.3)	25 (21.6)	464 (27.2)
1–5	565 (40.9)	60 (29.1)	27 (23.3)	652 (38.2)
6–10	234 (16.9)	41 (19.9)	27 (23.3)	302 (17.7)
> 10	191 (13.8)	59 (28.6)	37 (31.09)	287 (16.8)

Values are shown as n (%) unless otherwise noted

*SMI* Serious Mental Illness, *AP* Antipsychotics, *DM* Diabetes Mellitus, *CVD* Cardiovascular Disease, *CVR* Cardiovascular Risk, *FP* Family practice, *COPD* chronic obstructive pulmonary disease

## Results

**Fig. 1:**
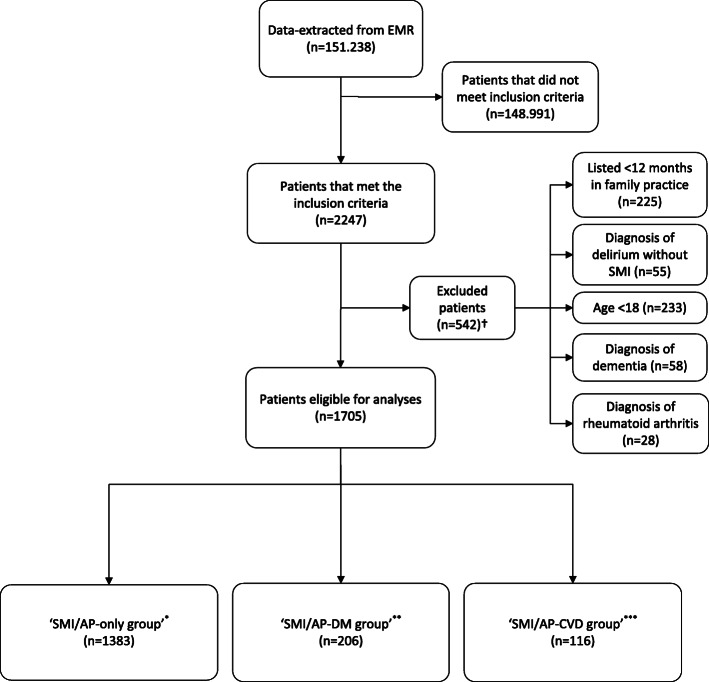
Flow chart of in- and exclusion of patients. ^*^ Patients with SMI/AP without another indication for yearly assessment of cardiovascular risk. ^**^ SMI/AP patients with additional diabetes. ^***^ SMI/AP patients with additional cardiovascular morbidity without known diabetes. † Some excluded patients fitted more than one exclusion criterion. EMR = Electronic Medical Records, SMI=Serious Mental Illness, DM = Diabetes Mellitus, CVD = Cardiovascular Disease

Of the 2247 SMI/AP patients (prevalence = 1.5%), 542 patients were excluded. Figure 1 shows the flow chart of in- and exclusion of patients.

Table 1 shows the demographic and clinical characteristics of our included patients. Of these, 14.7% patients were diagnosed with schizophrenia, 16.1% were diagnosed with an affective psychosis or bipolar disorder and 20.3% had a diagnosis of psychosis NOS. Of all 1150 patients using antipsychotics, 68.3% did not have any diagnoses concerning SMI in their medical records (*n* = 785). Quetiapine was the most commonly prescribed antipsychotic agent (20.1%). Of included patients 27.2% had less than one visit per year, while 16.8% had over 10 visits per year. The subgroup analysis (see Additional file 2) showed that patients with SMI more often had less than one visit while patients using AP without SMI more often had over 10 visits a year.

### CVD risk factor assessment

Table 2 presents the screening rate of CVR assessment for the three SMI groups. In 8.5% of the SMI/AP-only group, risk factors were adequately assessed. Logistic regression analysis resulted in ORs for adequate screening in the SMI/AP + DM and SMI/AP + CVD group when compared to the SMI/AP-only group of 24.6 (95% CI 17.3–35.1) and 4.2 (95% CI 2.7–6.6) respectively.

**Table 2:** Completeness of CVR screening for patients with SMI/AP and for subgroups with comorbid DM or CVD

Indication for CVR assessment	Insufficient	Moderate^a^	Adequate^b^	Odds ratio(95% CI)^c^
*SMI/AP-only group (*n = 1383)	90.2 (1247)	1.4 (19)	8.5 (117)	Reference group
*SMI/AP + DM group (n = 206)*	29.6 (61)	1.9 (4)	68.4 (141)	21.8(15.4–30.8)
*SMI/AP + CVD group (n = 116)*	68.1 (79)	5.2 (6)	26.7 (31)	4.3(2.8–6.6)

Values are shown in %(n) unless otherwise noted

*CVR* Cardiovascular Risk, *CI* Confidence Interval, *SMI* Serious Mental Illness, *AP* Antipsychotics, *DM* Diabetes Mellitus, *CVD* Cardiovascular Disease

^a^ BMI, smoking status and blood pressure were all recorded

^b^ BMI, smoking status, blood pressure, glucose and cholesterol/HDL ratio were all recorded

^c^ OR for an adequate & moderate screening rate

### Factors contributing to screening rate

Multivariate multilevel logistic analysis showed a high frequency of visits, age, AP use and a diagnosis of COPD were positively associated with an adequate screening rate in the SMI-only group (Table 3). SMI and AP are correlated and therefore could not be simultaneously part of the model. We chose for the variable with the most significant p-level, which was AP use.

**Table 3:** Patient characteristics associated with CVR screening for patients with SMI/AP who have no comorbid diagnosis of diabetes or CVD (*n* = 1383)

Factor	OR	95% CI
**Age**	1.05	1.036–1.055
**AP use +**	1.62	1.20–2.18
**COPD+**	2.8	1.87–4.31
***Number of visits FP/year***^***a***^		
> 10	2.24	1.65–3.03

Cardiovascular risk screening was considered to be performed if the assessment included at least BMI, smoking status and blood pressure

All significant variables identified by logistic regression analysis (*p* < 0.05) were included in this backwards stepwise regression procedure. ^a^Reference is ≤10 visits FP/year. *OR* Odds Ratio, *CI* Confidence Interval, *AP* antipsychotics, *COPD* Chronic Obstructive Pulmonary Disease, *FP* family practice

## Discussion

### Summary

Adequate screening for cardiovascular risk by FPs in patients with SMI/AP is very low (8.5%). In patients with additional comorbidity that require screening for CVR this was considerably higher, especially in patients with type 2 diabetes (68.4%). Screening increased with age, advancing number of visits, AP use and the presence of COPD. It was striking that in the majority of patients using AP, a diagnosis of SMI was not recorded in the EMR.

### Comparison with existing literature

The large group of AP users without a SMI diagnosis may indicate that patients use AP off-label. However, a part of this group consists of patients whose FP lacked information about the precise psychiatric disease or did not use the correct code. In addition, there are a few on-label indications for non-psychotic diseases, such as Quetiapine for unipolar, therapy-resistant depression. Other studies endorse the possibility of a high prevalence off-label AP use [20, 45–47].

The screening rate for CVR in patients with SMI/AP has been evaluated in several studies in different countries, resulting in a wide range of screening rates [27, 29, 31, 34, 48, 49]. This variation can be explained by differences in study population and methods and provides insights in factors to take into account when an intervention is considered. A study among patients in a US Medicaid program with newly prescribed AP found that 79.6% of the patients without DM were tested on glucose (non-fasting tests included) and 41.2% on lipids [34]. Failure to receive metabolic testing was most strongly associated with younger age, fewer chronic conditions and frequency of health care utilization regardless of the care setting (mental health care or primary care) [34]. Mangurian found that 73% of patients with SMI and DM were adequately tested in a 2 years’ time frame. This result is comparable with ours despite our broad inclusion of patients with SMI and those taking AP without SMI [48].

A Canadian study among patients from a community health centre specialized in patients with barriers to the healthcare system found adequate screening rates in over 70% of patients with SMI (*n* = 106) [49].

Intervention studies to improve the screening rate focused on financial compensation or organizational changes.

A primary care study in the UK showed that financial compensation for the task alone without organizational embedding is not enough. In this period of time, in the UK every primary care center received payment to provide care for patients with chronic conditions, including SMI, but only just over a fifth of patients with SMI received a full CVD screening, compared with 96% of patients with diabetes (OR = 90.4; 95% CI = 64.5–126.6, *p* < 0.01) [31]. Organizational changes are more promising. A large systematic review concluded that the presence and implementation of standard screening protocols, that were triggered by a diagnosis of SMI, may be promising avenues to ensure adequate diagnosis and screening of CVR assessment in patients with SMI [27].

The patients in our SMI/AP + DM group take part in a guideline-based integrated chronic care program due to having diabetes, resulting in almost 70% adequate screening. Although ‘high’, this is much lower than the screening rate for all patients with type 2 diabetes as a whole, exceeding 95% [50]. The National Diabetes Association (UK) reports on the proportion of people receiving the eight recommended care processes no difference between people with type 2 diabetes and SMI compared to people with type 2 diabetes alone (2016–2017) [51].

### Strengths and limitations

The main strength of our study is the size of the study sample and the broad inclusion of patients, based on diagnoses or on prescriptions of AP, which resulted in a realistic overview of the amount of psychiatric patients with an increased CVR in primary care. We therefore think the diversity of our study group is representative for primary care patients in daily practice in the Netherlands, which contributes to the validity and reliability of our findings.

Several limitations need to be mentioned as well. First, we studied whether or not FP’s screened patients with SMI/AP on CVR. The retrospective design offers limited insight on their motives. Second, we did not have access to patient records in mental health institutions, since we only used the EMRs from FPs. Consequently, it is imaginable that CVR was assessed in mental health care institutions and that our results are an underestimation of CVR screening. About half of the patients with SMI receive (additional) care from such institutions [52]. Third, it is important to keep in mind that by excluding patients who were listed for less than 12 months in a family practice (*n* = 225, 10% of all patients) there is a potential selection bias. Patients who switch FPs regularly might be homeless, uninsured or move frequently and consequently might not be screened at all. Their absence in our study can result in an overestimation of CVR screening. Forth, we think the small number of patients with abuse of alcohol and drugs is due to lack of capturing these data in the EMR of the FP. Therefore, the expected inverse relation with adequate screening could not be proven nor rejected.

## Conclusions

CVR screening of patients with SMI/AP poses a challenge.

FPs have a key position in the screening for CVR and an increasing role in the care for SMI patients. We recommend FPs to accurately record psychiatric diagnoses and be vigilant with off-label prescriptions. Standardized protocols to increase involvement of FPs create an opportunity to improve cardiovascular screening and re-evaluate AP use in patients without SMI diagnosis. Future studies should provide information concerning the best ingredients of a family physicians` chronic care program for patients with SMI/AP to improve their care.

## Supplementary information

**Additional file 1.**

**Additional file 2.**

## Data Availability

The datasets used and/or analyzed during the current study are available from the corresponding author on reasonable request.

## Abbreviations

SMI: serious mental illness
AP: antipsychotics
SMI/AP: SMI and/or AP
CVR: cardiovascular risk
DM: type 2 diabetes mellitus
CVD: cardiovascular disease
OR: odds ratio
CI: confidence interval
EMR: Electronic Medical Records
FP: family practitioner
ICPC: International Classification of Primary Care
ATC: Anatomical Therapeutic drug Chemical classification
COPD: chronic obstructive pulmonary disease
