# Supplementary material for: Cardiovascular risk screening of patients with serious mental illness or use of antipsychotics in family practice
Source: BMC Fam Pract. 2020 Jul 29;21:153. doi: 10.1186/s12875-020-01225-7 (PMC7391510; doi:10.1186/s12875-020-01225-7)
Supplement: Supplementary file 1 — Additional file 1. [file 12875_2020_1225_MOESM1_ESM.docx]

## Additional file 1

Appendix A1- Included diagnostic assays

| **Preferred assay** | **Also included** |
| --- | --- |
| BMI | Body weight, length, waist circumference |
| Systolic bloodpressure | Diastolic bloodpressure |
| eGFR | Creatinin, albuminuria, albumin/creatinin ratio |
| Smoking status |  |
| Fasting glucose | Non-fasting glucose, HbA1c |
| Total cholesterol/HDL ratio | LDL, total cholesterol, HDL, triglycerides |
| Use of alcohol |  |
| Family history of cardiovascular disease |  |
| Physical activity |  |
| Dietary Intake |  |

Appendix A2- ICPC codes

| **Description or Diagnosis** | **ICPC code** |
| --- | --- |
|  |  |
| **Psychological diseases** |  |
| Schizophrenia | P72, P72.01 |
| Affective psychosis, bipolar disorder | P73, P73.02 |
| Psychosis NOS/other | P98 |
| Dementia | P70, P70.01, P70.02 |
| Delirium tremens | P15.02 |
| Organic psychosis | P71, P71.04 |
| Chronic alcohol abuse | P15, P15.01, 15.03, P15.05, P15.06 |
| Acute alcohol abuse | P16 |
| Tobacco abuse | P17 |
| Medication abuse | P18 |
| Drug abuse | P19, P19.01, P19.02 |
|  |  |
| **Cardiovascular diseases** |  |
| Stroke | K90, K90.01, K90.02, K90.03 |
| Ischemic heart disease w. angina | K74, K74.01, K74.02 |
| Acute Myocardial Infarction | K75 |
| Ischemic heart disease w/o angina | K76, K76.01, K76.02 |
| Transient cerebral ischemia | K89 |
| Intermittent claudication | K92.01 |
| Aneurysm aorta | K99.01 |
|  |  |
| **Diseases associated with an increased risk of cardiovascular disease or indication for yearly risk assessment** |  |
| Rheumatoid arthritis | L88, L88.01, L88.02 |
| Diabetes Mellitus | T90, T90.01, T90.02 |
| Chronic obstructive pulmonary disease | R95 |
|  |  |
| **Social issues** |  |
| Poverty/financial problem | Z01 |
| Housing/neighborhood problem | Z03, Z03.01, Z03.02, Z03.03 |
| Loneliness | Z04.03 |
| Unemployment problem | Z06 |
| Analphabetism | Z07.01 |
| Social welfare problem | Z08, Z08.01, Z08.02 |
| Health care system problem | Z10 |
| Relationship problem with partner | Z12, Z12.01, Z12.02 |
| Partner’s behavior problem | Z13, Z13.01, Z13.02, Z13.03 |
| Child neglect | Z16.02 |
| Limited social function | Z28 |

Appendix A3- ATC codes and grouping of medication

| **Medicine** | **ATC code** |
| --- | --- |
|  |  |
| **Antipsychotics** |  |
| Chlorpromazine | N05AA01 |
| Levomepromazine | N05AA02 |
| Fluphenazine | N05AB02 |
| Perphenazine | N05AB03 |
| Periciazine | N05AC01 |
| Haloperidol | N05AD01 |
| Pipamperone | N05AD05 |
| Bromperidol | N05AD06 |
| Sertindole | N05AE03 |
| Flupentixol | N05AF01 |
| Chlorprothixene | N05AF03 |
| Zuclophenthixol | N05AF05 |
| Fluspirilene | N05AG01 |
| Pimozide | N05AG02 |
| Penfluridol | N05AG03 |
| Clozapine | N05AH02 |
| Olanzapine | N05AH03 |
| Quetiapine | N05AH04 |
| Sulpiride | N05AL01 |
| Tiapride | N05AL03 |
| Lithium | N05AN01 |
| Risperidone | N05AX08 |
| Aripirazole | N05AX12 |
| Paliperidone | N05AX13 |
|  |  |
| **Antidepressants** | N06A |
