# Supplementary material for: Cardiovascular risk screening of patients with serious mental illness or use of antipsychotics in family practice
Source: BMC Fam Pract. 2020 Jul 29;21:153. doi: 10.1186/s12875-020-01225-7 (PMC7391510; doi:10.1186/s12875-020-01225-7)
Supplement: Supplementary file 2 — Additional file 2. [file 12875_2020_1225_MOESM2_ESM.docx]

**Addendum 1**

In this addendum, we analyzed whether our results differed between two groups: patients with a SMI diagnosis and patients who use AP without a SMI diagnosis. We compared the patient characteristics between these two groups (Table A1), assessed the completeness of their CVR screenings (Table A2), and explored the factors associated with adequate screening (Table A3) separately for these two groups.

Table A1. Comparison of patient characteristics between the patients with SMI and the patients using AP without a recorded SMI diagnosis.

|  | *Patients with SMI (n = 689)* | *Patients using AP without recorded SMI (n = 630)* | *P-value* |
| --- | --- | --- | --- |
| **Mean age**, years (SD) | 43 (14.5) | 44 (14.7) | 0.34 |
| **Sex**, female | 323 (46.9) | 359 (57.0) | <0.001* |
| **Antidepressants** | 140 (20.3) | 381 (60.5) | <0.001* |
| **CVR-lowering medication** | 126 (18.3) | 146 (23.2) | 0.028* |
| **COPD** | 23 (3.3) | 35 (5.6) | 0.05* |
| **Alcohol abuse** | 37 (5.4) | 29 (4.6) | 0.52 |
| **Smoker** | 102 (14.8) | 125 (19.8) | 0.02* |
| **Drug abuse** | 55 (8.0) | 45 (7.1) | 0.56 |
| **Number of FP visits/year** |  |  | <0.001* |
| 0 | 221 (32.1) | 156 (24.8) |  |
| 1–5 | 288 (41.8) | 245 (38.9) |  |
| 6–10 | 107 (15.5) | 118 (18.7) |  |
| >10 | 73 (10.6) | 111 (17.6) |  |

Values are shown as n (%) unless otherwise noted.

SMI: serious mental illness; AP: antipsychotics; CVR: cardiovascular risk; COPD: chronic obstructive pulmonary disease; FP: family practice.

Table A1 shows that the two groups differ in several aspects. The percentages of women, patients taking antidepressants or CVR-lowering medication, patients with a diagnosis of COPD, and patients who use tobacco, as well as the number of FP visits, are all significantly lower in the group of patients with SMI than in the group of patients using AP without a recorded SMI diagnosis.

Table A2. Completeness of CVR screening for patients with SMI and for patients using AP without a recorded SMI, and for subgroups with comorbid DM or CVD.

| **Indication for CVR assessment** | *Insufficient* | *Moderate^^^/Adequate^#^* | *Odds ratio (95%CI)^*^* |
| --- | --- | --- | --- |
| SMI (n = 689) | 92.7 (639) | 7.3 (50) | Reference group |
| SMI+DM (n = 97) | 39.2 (38) | 60.8 (59) | 19.8 (12.1–32.7) |
| SMI+CVD (n = 48) | 72.9 (35) | 27.1 (13) | 4.7 (2.4–9.5) |
| AP use without SMI (n = 630) | 88.1 (555) | 9.7 (61) | Reference group |
| AP+DM (n = 95) | 22.1 (21) | 77.9 (74) | 26.1 (15.2–44.8) |
| AP+CVD (n = 60) | 65.0 (39) | 35.0 (21) | 4.0 (2.2–7.1) |

Values are shown in % (n) unless otherwise noted.

^ BMI, smoking status, and blood pressure were all recorded (CVR screening without the need of a blood sample).

# BMI, smoking status, blood pressure, glucose and cholesterol/HDL ratio were all recorded.

* Odds ratio for an adequate and moderate screening rate.

CVR: cardiovascular risk; CI: confidence interval; SMI: serious mental illness; AP: antipsychotics; DM: diabetes mellitus; CVD: cardiovascular disease.

Table 2 reveals that the rate of adequate CVR screening by FPs is very low in both groups (7.3% for the group of patients with SMI, 9.7% for the group of patients using AP without SMI, and 8.5% for both groups combined). In patients with a comorbidity that requires CVR screening, the rate of adequate screening was considerably higher, especially in patients with type 2 diabetes (60.8%, 77.9%, and 68.4% for the SMI group, the AP group, and both groups combined, respectively). A comparison between Table 2 of the main article (OR=21.8 and 4.3, respectively, for SMI/AP+DM and SMI/AP+CVD) and Table A2 in this Addendum (OR=19.8 and 4.7 for SMI+DM and SMI+CVD, respectively, and 26.1 and 4.0 for AP without SMI but with DM or CVD, respectively) shows that the OR of the rate of screening was only slightly affected by the division of the SMI/AP group of patients into separate groups. These results therefore support the conclusion of the main article, which states that the rate adequate CVR screening by FPs in patients with SMI or those using AP is very low, whereas patients with additional comorbidities that require CVR screening have a considerably higher screening rate.

Table A3. Factors most associated with CVR screening for patients with SMI and patients using AP without a recorded SMI diagnosis who have no comorbid diagnosis of diabetes or CVD.

| **Group** | | *Factor* | *OR* | | *95% CI* | |
| --- | --- | --- | --- | --- | --- | --- |
| SMI | | Age | 1.04 | | 1.02–1.06 | |
| SMI | | COPD present | 5.62 | | 2.12–14.94 | |
| SMI | | Number of FP visits/year* |  | |  | |
|  |  | >10 | 3.65 | | 1.83–7.28 | |
| AP without SMI | Age | | | 1.04 | | 1.02–1.06 |
| AP without SMI | COPD present | | | 2.36 | | 1.05–5.29 |
| AP without SMI | Number of FP visits/year* | | |  | |  |
|  | >10 | | | 1.89 | | 1.07–3.37 |

Cardiovascular risk screening was considered to be adequately performed if the assessment included at least BMI, smoking status, and blood pressure.

All significant variables identified using the logistic regression analysis (p < 0.05) were included in this backwards stepwise regression procedure.

*Reference is 0 FP visits/year

OR: odds ratio; CI: confidence interval; AP: antipsychotics; COPD: chronic obstructive pulmonary disease.

The ORs of the factors age, COPD, and >10 FP visits/year shown in Table 3 are comparable with those in Table 3 of the main article (respectively, 1.05; 2.8; 2.24). This suggests that it is valid to combine the patients with SMI group with the patients using AP without SMI.
